# Functional analysis of splice variant expression of *MADS AFFECTING FLOWERING 2* of *Arabidopsis thaliana*

**DOI:** 10.1007/s11103-012-9982-2

**Published:** 2012-10-31

**Authors:** Sarah Marie Rosloski, Anandita Singh, Sathya Sheela Jali, Sureshkumar Balasubramanian, Detlef Weigel, Vojislava Grbic

**Affiliations:** 1 Department of Biology, Western University, London, ON N6A 5B8 Canada; 2 Department of Molecular Biology, Max-Planck Institute for Developmental Biology, 72076 Tübingen, Germany; 3 Present Address: Department of Biotechnology, Faculty of Applied Science, TERI University, 10 Institutional Area, Vasant Kunj, New Delhi, 110070 India; 4 Present Address: School of Biological Sciences, Monash University, Clayton, VIC 3800 Australia

**Keywords:** *Arabidopsis thaliana*, Flowering time, Alternative splicing, Response to cold, *MAF2*

## Abstract

The *MADS*-*AFFECTING FLOWERING 2* (*MAF2*) gene of *Arabidopsis thaliana* has been characterized as a repressor of flowering. The molecular basis of *MAF2* gene function and role of alternative *MAF2* transcripts in flowering time modulation is not understood. *MAF2* splice variant expression was quantified in cold-acclimated plants by quantitative RT-PCR. Cold influenced the abundance of splice variants and prompted a functional study of splice forms. Individual variants were overexpressed in the Col background and were assayed for their ability to delay flowering. Overexpression of *MAF2* variants 2 and 4 had limited effect on flowering time. Overexpression of *MAF2* splice variant 1 resulted in early flowering and affected the expression of the endogenous *MAF2* gene and its paralogues, confounding functional assessment. In the Ll-2 Arabidopsis accession, a *MAF2*, *MAF3*, *MAF4* and *FLC* null line, *MAF2* var1 was consistent in its effect on reproductive delay under ambient and reduced temperatures, indicating that it acts as a repressor of flowering.

**Electronic supplementary material:**

The online version of this article (doi:10.1007/s11103-012-9982-2) contains supplementary material, which is available to authorized users.

## Introduction

The number of genes discovered in early genome sequencing projects was lower than anticipated considering the apparent complexity of eukaryotic organisms (Rubin et al. 2000; International Human Genome Sequencing Consortium 2001). However, a small gene set may result in complex phenotypes if genes produce multiple products with diverse functions (Pesole 2008). Alternative splicing is one of the mechanisms that can expand the functional diversity of genes (Modrek and Lee 2002; Reddy 2007). Alternative splicing operates through selection of variable donor and acceptor sites during pre-mRNA processing and can result in a suite of divergent mRNAs produced from a single gene (Sharp 1994). Alternative splicing can instigate a spectrum of functional outcomes that may considerably complicate the assignment of gene function.

In plants, alternative splicing is wide-spread. In *Arabidopsis* and rice, it affects 32.5 and 23.5 % of genes, respectively (Campbell et al. 2006). Many alternative splicing events are shared between these species, suggesting a functional role for splice product variation (Wang et al. 2008). Alternative splicing is particularly apparent during environmental stress responses (Iida et al. 2004; Robinson and Parkin 2008). Splice site decisions are sensitive to salt, temperature, wounding, metal contamination of the soil, light levels and stress hormones (Simpson et al. 2008; Bove et al. 2008; Palusa et al. 2007, Iida et al. 2004; Marrs and Walbot 1997; Robinson and Parkin 2008). Thus, alternative splicing may be an additional mechanism of sensing or responding to environmental cues. Despite the importance of alternative splicing, most reports only describe alternative gene products but do not elucidate the function of alternative transcripts (Reddy 2007).

The initiation of flowering is crucial for plant reproductive success. Plants integrate both intrinsic developmental and environmental cues such as day-length and temperature during the transition from the vegetative to reproductive development and some of these decisions are influenced by components of the spliceosome or are splice variant specific (Xing et al. 2008; Quesada et al. 2003). Fifty-nine out of the approximately 80 genes in Arabidopsis that affect timing of flowering display transcripts with splice or poly-adenylation site variation and 24 of these genes are known to be involved in pre-mRNA processing (Terzi and Simpson 2008; Herr et al. 2006; Lopato et al. 1999; Wang and Brendel 2006; Wang et al. 2007; Noh et al. 2004). Though flowering time control is influenced at multiple levels, alternative splicing may provide an additional responsive sensor of environmental changes.

*MADS*-*AFFECTING FLOWERING 2* (*MAF2*) is a member of a tandem quadruplication of MADS-box transcription factor genes *MADS*-*AFFECTING FLOWERING 2*–*5* (*MAF2*, *MAF3*, *MAF4*, *MAF5*) in Arabidopsis. These genes are homologous to *FLOWERING LOCUS M* (*FLM/MAF1*) and *FLOWERING LOCUS C* (*FLC*). Genes from the *FLC/MAF* clade are major determinants of natural flowering time variation, as Quantitative Trait Loci (QTLs) at *FLC*, *MAF1*, *MAF2*–*5* account for 19, 15 and 15 % of natural variation in flowering time in the studied populations, respectively (Salome et al. 2011). All six genes display between 2 and 5 unique splice variants and expression of all genes is responsive to temperature (Sung et al. 2006; Caicedo et al. 2004; Ratcliffe et al. 2003; Micheals and Amasino 1999). The effect of temperature on gene expression, downstream pathways and strength of the phenotypic effect has diverged between the best known clade members *FLC*, *FLM/MAF1* and *MAF2* (reviewed in Alexandre and Hennig 2008). Alteration of the splice variant profile by temperature (at 27 °C) has been reported for *FLM/MAF1* and *MAF2*, though the effect has not been studied quantitatively nor is the qualitative function of alternative splice variants known (Balasubramanian et al. 2006). Neither *FLM/MAF1* nor *MAF2* major alternative variants are predicted to be targeted by the Nonsense Mediated Decay pathway and therefore may be translated (Severing et al. 2012). *FLM/MAF1* variants display mutually exclusive exon and intron retentions and the *MAF2* alternative variants display an alternative acceptor site and premature transcript termination (Severing et al. 2012, Ratcliffe et al. 2003). Unlike *FLM/MAF1*, the splice variant profile of *MAF2* is conserved in *Brassica napus* and *Brassica rapa* orthologues, suggesting a possible functional relevance (Severing et al. 2012). Also, unlike *FLM/MAF1*, the two major *MAF2* splice variants, *MAF2* variant (var) 1 and 2, are expressed distinctly and strongly, increasing the likelihood of functional relevance and rendering them more tractable to study (Balasubramanian et al. 2006). Therefore, *MAF2* is a good candidate to understand the role of alternative splicing in the determination of flowering time, though the conclusions made for this gene may not be transferable to other clade members.

Though the effect of temperature on the *MAF2* splice variant profile has been reported at moderately high temperatures, the effect of *MAF2* on plant phenotype is best understood at low, 4 °C, and optimal growth temperatures, 21 °C. Study of *MAF2* function showed that *maf2* plants that are lacking *MAF2* expression displayed a weak acceleration of flowering when grown at 21 °C (Ratcliffe et al. 2003). *MAF2* had a stronger capability to delay flowering after a short, 16–21 day, cold treatment, suggesting that *MAF2* prevents flowering in the event of autumnal temperature fluctuations that might be misinterpreted as the arrival of spring (Ratcliffe et al. 2003). Unlike plants lacking *FLC* function, *maf2* plants lacking *MAF2* function exposed to a long, 85-day cold treatment flowered similarly to controls (Ratcliffe et al. 2003). Notwithstanding, it is unclear if a complete knock-out of transcription at an alternatively spliced gene can adequately explain gene function.

In this study, we show that expression levels of the *MAF2* splice variants are temperature-dependent. During the course of the cold treatment the accumulation of *MAF2* var1, the transcript form predicted to generate the full-length protein, is maintained, and the abundance of *MAF2* var2 transcript, predicted to encode the truncated MAF2 protein decreases. Phenotypic analysis of gain-of-function transgenic plants revealed that the *MAF2* var1 has the most consistent effect on repression of flowering, indicating that it acts as a repressor of flowering.

## Materials and methods

### Plant material

The seed of Col and Ll-2 *Arabidopsis* accessions were acquired from the *Arabidopsis* Biological Resource Center (ABRC). The seed of the L*er* accession was acquired from X. Chen (Chen and Meyerowitz 1999) and *flc*-*3* from R. Amasino (Micheals and Amasino 1999). Col and L*er* have wild-type *MAF2* alleles, however, *FLC* and *MAF2* expression is not detectable in Ll-2 (Lempe et al. 2005; Rosloski et al. 2010). Plants were grown at 22 °C under a long day (LD), 16 h light/8 h dark, or a short day (SD) photoperiod, 8 h light/16 h dark, under 100–150 μmol/m^2^ s cool-white fluorescent lights. All seedlings were given at least a 3-day stratification period prior to germination. Cold treatment was administered by growing seedlings on half-strength MS media in petri plates in a temperature-controlled 4 °C incubator under low light conditions 16 days before shifting to LD or SD conditions. T_1_ transgenic lines overexpressing individual *MAF2* transcripts in Col and *flc*-3 backgrounds were initially grown at 4 °C for 10 days and were then transferred to LD conditions at 16 °C to enhance potential differences in flowering time. Tissue was collected and bulked from 5 T_1_ transgenic plants and assayed in triplicate to assess, on average, if plants were expressing the transgene using qRT-PCR and primers 25 and 26 (Supplemental Table 1). A spectrum of flowering phenotypes from T_1_ transgenic lines in the Col background was used to derive T_4_ transgenic plants. T_4_ transgenic lines with a single copy of transgenes were selected.

### Generation of gain-of-function transgenic plants

cDNA clones corresponding to splice variants AY231441 (var1, full-length), AY231442 (var2), AY231444 (var4) and the full-length genomic construct of *MAF2* (*MAF2*g) were cloned into the *Sma*I site of the pCHF3 binary vector, and were placed under control of the strong constitutive 35S promoter (Fig. 2a). The Col, *flc*-*3* and Ll-2 accessions were transformed using the floral dip method (Clough and Bent 1998). Transformants were selected on the half-strength MS media supplemented with 50 mg/L kanamycin.

### Analysis of the effect of cold on gene expression

For the comparative analysis of temperature on *MAF2* expression, sterile seeds were stratified in the dark for 3 days at 4 °C on half-strength MS media in sealed Petri plates. As Col plants grown in LD conditions are committed to reproductive development at around 7 days after germination (dag) and as we are interested in the role of temperature-sensitive *MAF2* splice variant expression in the determination of flowering time, we cold-treated plants 3 dag under a non-inductive SD photoperiod (Bradley et al. 1997). Seeds were transferred to a 21 °C chamber with a SD photoperiod for 3 days. At this time, seedlings had emerged from the seed coat without extensive expansion of the cotyledons. These seedlings were sampled and are referred to as day 0. The petri plates were then either transferred to a 4 °C, SD growth chamber or remained at 21 °C SD for 3, 6, 12 and 18 days. Seedlings in the 4 °C treatment remained arrested at the cotyledon stage and did not display expansion of true leaves. Seedlings in the 21 °C treatment developed true leaves as the experiment progressed and displayed ~4 small leaves by day 12. Tissue for cold-treated seedlings was collected at 4 °C and all tissues were frozen immediately in liquid nitrogen prior to RNA extraction.

### Analysis of the effect of cold on flowering time

T_2_ seeds were collected from plants overexpressing *MAF2* genomic or var1 constructs in Ll-2, as analysed in Fig. 5. Cold-treated seeds were sterilized and plated on half-strength MS media supplemented with 50 mg/L kanamycin and placed in a 4 °C chamber for 16 days. On the 13th day, another set of seeds was sterilized, plated on half-strength MS media supplemented with 50 mg/L kanamycin and stratified for 3 days at 4 °C. Both 16 day cold-treated and 3 day stratified seedlings were planted simultaneously in at least 3 replicates of 4 plants per pot and grown under SD conditions, as previously described. Over half of Ll-2 T_2_ plants expressing both *MAF2*g and *MAF2* var1 transgenes displayed severe leaf deformities and failed to maintain the apical meristem after germination. Only plants that were phenotypically normal and attained reproductive development were analysed. Time to flowering was determined by counting rosettes leaves (RLN) produced prior to bolting.

### Analysis of gene expression

RNA was extracted using the RNeasy Plant RNA Extraction Kit (Qiagen) as per manufacturer’s instructions. For semi-quantitative RT-PCR (sqRT-PCR), the number of PCR cycles was determined to reflect the log phase of product accumulation for various primer combinations (see Supplemental Table 1). Quantitative RT-PCR (qRT-PCR) primer combinations were optimized for melting temperature and MgCl_2_ concentration. Real-time qRT-PCR amplification of *MAF2* var1 and *MAF2* var2 using primers 15 and 16, or 17 and 18, respectively, and reaction conditions are found in Supplemental Table 1. qRT-PCR was conducted on the Corbett Rotor Gene 3000 using two biological replicates and at least three technical replicates. Relative target amplification was expressed using the 2T(-Delta Delta C) method (Livak and Schmittgen 2001). Expression of the *TUBULIN2* (*TUB*) was monitored as a loading control in all semi-quantitative and quantitative RT-PCR experiments using primers and reaction conditions specified in Supplemental Table 1.

### Statistical analysis

Statistical analysis was conducted by ANOVA in the program PAST version 1.81 (http://folk.uio.no/ohammer/past/index.html).

## Results

### *MAF2* gene expression is affected by the ambient temperature

To understand the regulation of *MAF2* splice variation by temperature, Col and Ler seeds were germinated for 3 days at 21 °C and seedlings were then transferred to either 4 or 21 °C. Seedlings were collected for RNA extraction from both temperature treatments at 3, 6, 12 and 18 days after the transfer. Seedlings were kept under a short-day (SD) photoperiod to insure that all were developing vegetatively throughout the duration of the experiment.

**Fig. 1:**
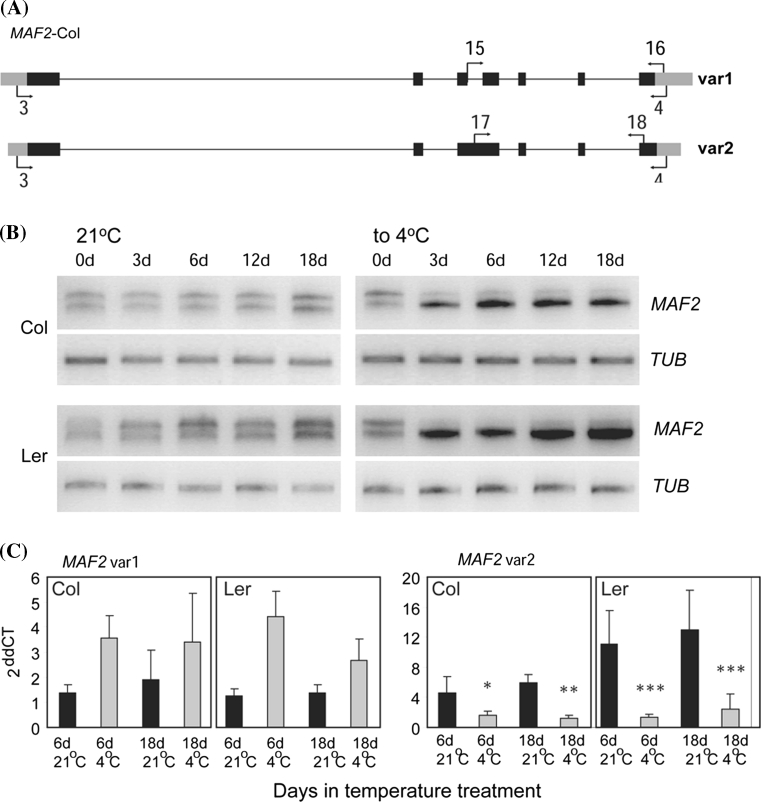
Temperature influences the splice variant profile of *MAF2*. **a** Graphic representation of two *MAF2*-Col/Ler splice variants and position of primers used (*arrows*). *Grey boxes*, UTR; *black boxes*, exons; *black lines*, introns. **b** Representative semi-quantitative RT-PCR of *MAF2* var1 and var2 transcripts amplified by using primers 3 and 4. The *upper band* corresponds to *MAF2* var2 and the *lower band* to *MAF2* var1. 0d (days) corresponds to 3-day-old seedlings grown at 21 °C. Subsequently, control seedlings remained at 21 °C, and cold-treated seedlings were transferred to 4 °C for 3, 6, 12 and 18 days. *TUB* amplification was used as a loading control. **c** Comparison between the expression of *MAF2* var1 and *MAF2* var2 in Col and Ler seedlings grown at 21 °C (*black bars*) and 4 °C (*grey bars*) at 6 and 18 days after the transfer of seedlings to 4 °C. Primers 15 and 16, and 17 and 18 were used for quantitative RT-PCR analysis of *MAF2* var1 and *MAF2* var2 expression, respectively. *TUB* amplification was used as a loading control. Statistical analysis was conducted on two biological replicates and three technical replicates by ANOVA. *p < 0.05; **p < 0.01; ***p < 0.0001

Temperature treatments altered the relative expression levels of *MAF2* splice variants compared to control seedlings. Using sqRT-PCR, the same pattern is obtained in both Col and Ler, characterized by two bands corresponding to the lower band *MAF2* var1 (Genbank accession AY231441, Ratcliffe et al. 2003), and the upper band corresponding to *MAF2* var2 (Genbank accession AY231442, Ratcliffe et al. 2003), (Fig. 1a, b; Supplemental Table 1). Another reported *MAF2* splice variant, *MAF2* var 4 (Genbank accession AY231444, Ratcliffe et al. 2003), consisting of only the short, MADS-Box DNA-binding domain, cannot be specifically amplified because of the highly conserved nature of this region preventing us from assessing the effect of temperature on this particular transcript. *MAF2* var1 is predicted to encode a full-length, MADS-box transcription factor, while *MAF2* var2 encodes a truncated protein that is predicted to have a MADS-box DNA binding domain but lacks the K-box protein interaction domain (Supplemental Fig. 1). The two *MAF2* variants remained at an equivalent relative staining intensity for the entire time series in seedlings grown at 21 °C (*MAF2*, to 21 °C, Fig. 1b). In contrast to controls, the splice variant profiles of seedlings transferred to 4 °C changed. The expression of *MAF2* var1 increased and the expression of *MAF2* var2 decreased (*MAF2*, to 4 °C, Fig. 1b). To obtain a quantitative measure of these changes, we performed qRT-PCR (Fig. 1c). This analysis showed that cold significantly decreases the abundance of the *MAF2* var2, predicted to encode a truncated MAF2 protein. In contrast, the expression of *MAF2* var1, predicted to encode the full-length MAF2 protein, has been maintained at a high level. Change in the abundance of these two transcripts during the short period of cold prompted us to address their roles in the regulation of flowering time.

### Overexpression of *MAF2* splice variants in the Col background

**Fig. 2:**
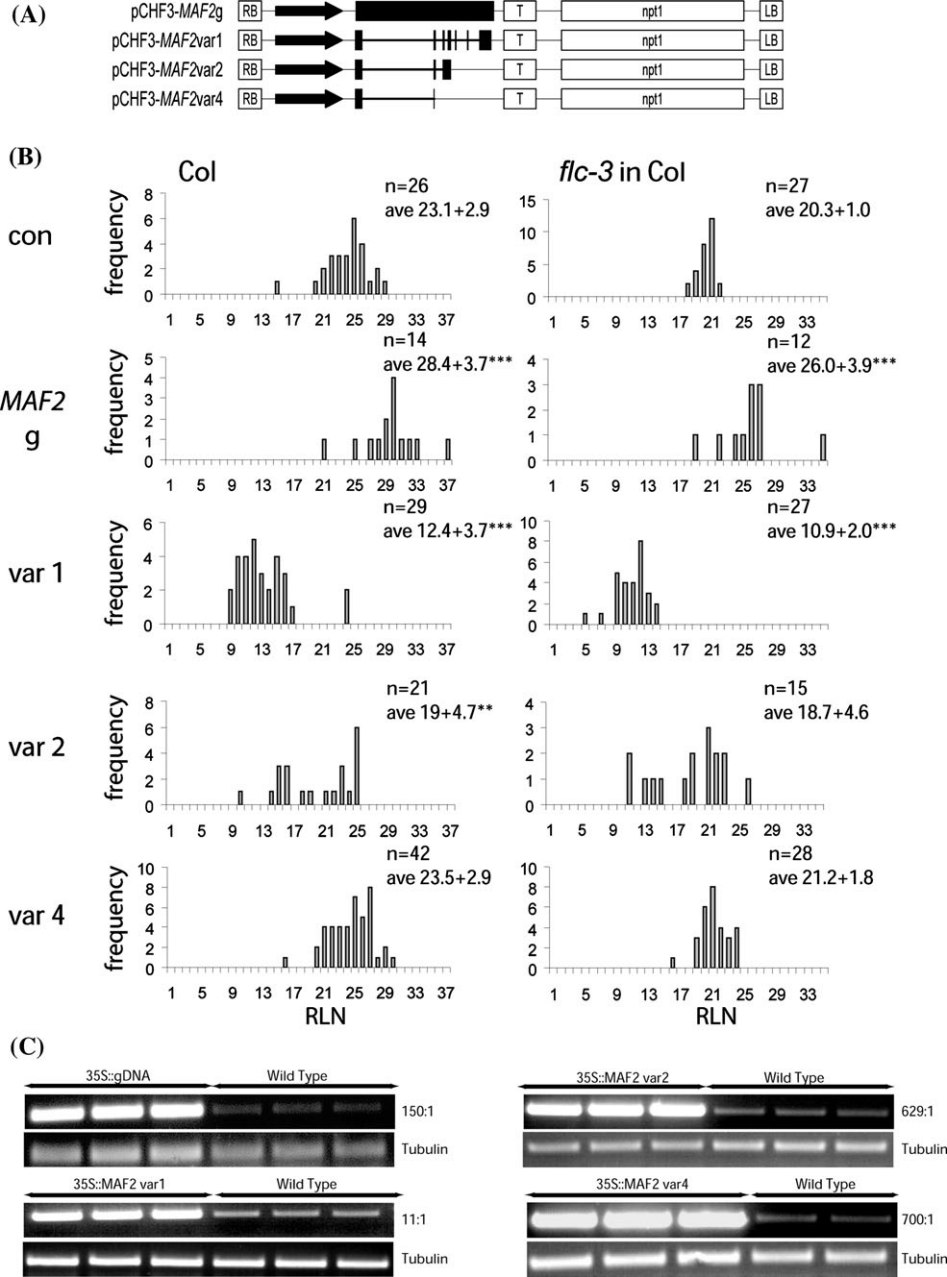
Flowering time frequency distribution of T_1_ transgenic lines over-expressing individual *MAF2* transcripts in Col and *flc*-*3* backgrounds. **a** Transgene constructs used to create Col and *flc*-3 plants constitutively expressing *MAF2* genomic clone, *MAF2* var1, *MAF2* var2 and *MAF2* var4. Each transgene consisted of constitutive 35S promoter (*black arrow*), a *MAF2* transgene sequence (depicted as *black boxes*) and a transcriptional terminator (T). A genomic construct expressed both exons and introns (shown as a *continuous black box*), while *MAF2* var1, *MAF2* var2 and *MAF2* var4 expressed corresponding open reading frames encoding specific variants (representative exons included in the transgene sequence are shown as *black boxes*). **b** Frequency distribution of flowering time in transgenic plants expressing an empty vector control (con), the entire *MAF2* genomic fragment (*MAF2*g), *MAF2* var1 (var 1), *MAF2* var2 (var 2) and *MAF2* var4 (var4). The average rosette leaf number, RLN ± SD and results of an ANOVA test indicating significant differences from the control at * <0.05, ** <0.01, *** <0.001 are shown in the *right hand corner* of each graph. **c** Transgene expression by qRT-PCR in overexpression lines depicted in b. Tissue from 5 T_1_ plants was collected and bulked for the analysis in three technical replicates, reflecting an average transgene expression within the population. The ratio of average expression in the overexpression pool relative to the control pool is shown on the *right* of each gel. Amplification represents total expression from the endogenous *MAF2* gene and the transgene using primers 5 and 20, and 25 and 26, respectively (Supplemental Table 1)

To address whether alternative splice variants produced by *MAF2* differ in their ability to modulate flowering time, we generated transgenic plants expressing the *MAF2* genomic clone (*MAF2*g), *MAF2* var1, *MAF2* var2 and *MAF2* var4 under the transcriptional control of the constitutive 35S promoter in Col and *flc*-*3* backgrounds (Fig. 2). Overexpression of all constructs in T_1_ plants was verified and resulted in a similar flowering time distribution patterns for Col and *flc*-*3* transgenic populations (Fig. 2b, c; Supplemental Table 2). Overexpression of *MAF2*g delayed flowering, consistent with the proposed role for *MAF2* as a floral repressor. However, no individual splice variant was capable of conferring a late-flowering phenotype. Instead, *MAF2* var1 overexpression resulted in pronounced early flowering in both Col and *flc*-*3* backgrounds. *MAF2* var2 produced slightly early flowering plants in Col and had no effect on flowering time in *flc*-*3* in Col, while *MAF2* var4 had no effect on flowering time in either Col or *flc*-*3*. As such, results of overexpression of *MAF2* splice variants in Col do not conform to our expectation that any variant individually encodes a floral repressor but that they act either as a powerful accelerator of flowering (*MAF2* var1) or have little or no effect on flowering (*MAF2* var2 and *MAF2* var4).

**Fig. 3:**
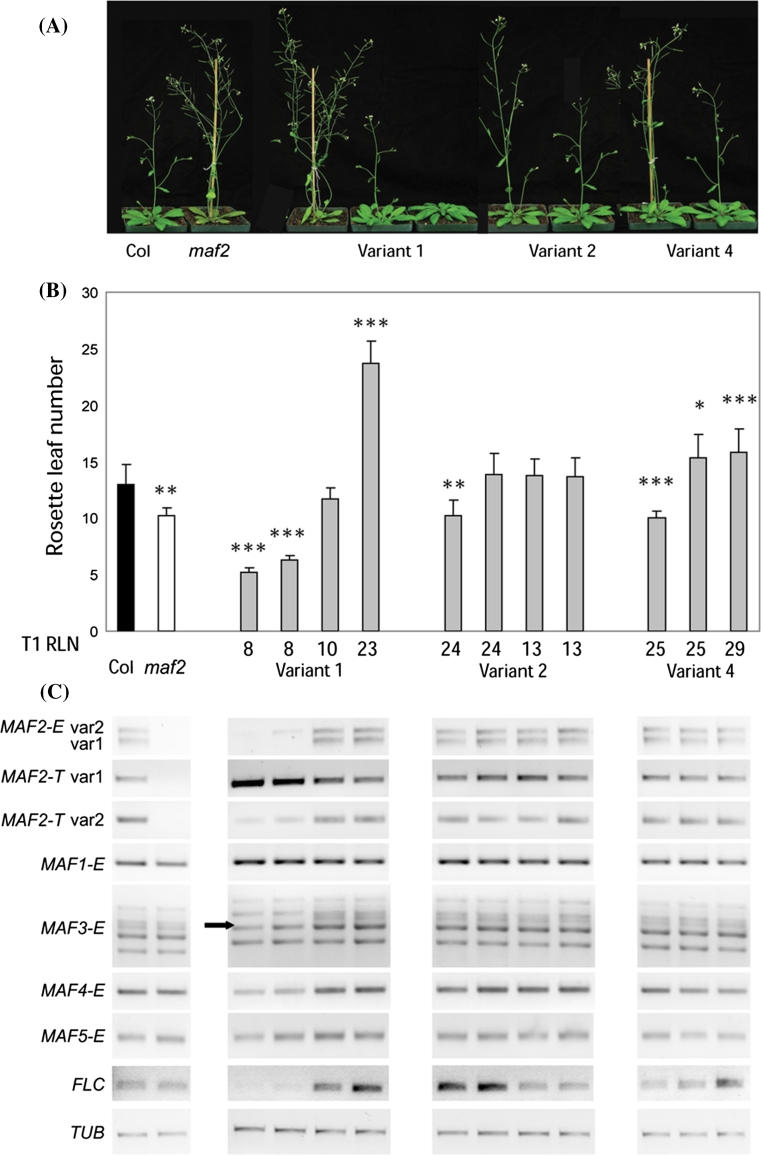
The effect of overexpression of *MAF2* splice variants on flowering time and the expression of endogenous *MAF2*, *MAF3*, *MAF4* and *FLC* genes in T_4_ lines in Col. **a** Representative plants depicting the range of flowering phenotypes observed in T_4_ lines grown under LD photoperiod at 22 °C. **b** Flowering time of T_4_ lines. Flowering time is expressed as the rosette leaf number (RLN) at flowering. An *asterisk* indicates significant difference relative to the control (an empty vector in Col). Significant difference from the Col control is indicated by * <0.05, ** <0.01, *** <0.001. T_1_ RLN, depicts the flowering time of progenitor T_1_ lines of analysed T_4_ lines. The flowering times were determined on 12 plants per genotype. **c** Representative semi-quantitative RT-PCR of endogenous *MAF2* gene expression (*MAF2*-E, primers 3 and 4), total *MAF2* gene expression (*MAF2*-T, endogenous and transgene *MAF2* expression, primers 5 and 6), *MAF1/FLM* (primers 1 and 2), *MAF3* (primers 7 and 8), *MAF4* (primers 9 and 10), *MAF5* (primers 11 and 12) and *FLC* (primers 13 and 14) genes is shown *below* the flowering time data for each T_4_ line. The *arrow* *adjacent* to the *MAF3*-E points to the *MAF3* transcript that is down-regulated in the early flowering T_4_ line overexpressing the *MAF2* var1. *TUB* expression was used as a loading control. Primer sequences are reported in Supplemental Table 1

To substantiate these unexpected results, we analyzed the flowering time and transgene expression of T_4_ homozygous, single-insert progeny that represent the spectrum of flowering times within each T_1_ *MAF2* variant class (Fig. 3). The early flowering 35S:*MAF2* var1 T_4_ lines were the only lines with high expression of the transgene (transgene expression could not be uniquely determined, but can be inferred from the total *MAF2* expression (*MAF2*-T, which reflects the expression from both the endogenous *MAF2* gene and the transgene) and endogenous *MAF2* expression (*MAF2*-E)), Fig. 3c. These plants flowered earlier than the *maf2* null allele (Fig. 2b, *35S:MAF2* var1, n = 12, flowered after forming 5.2 ± 0.40 and 6.3 ± 0.45 leaves; *maf2*, n = 12, flowered after forming 10.3 ± 0.65 leaves). Consistent with this pronounced early flowering phenotype we found reduced expression of the endogenous *MAF2* var1 and var2 splice forms (Fig. 3c). These lines also showed reduced expression of the *FLC* gene. Clear resolution of the *MAF3* expression profile revealed that it is changed in these early flowering lines as well. A single *MAF3* band was absent from the early flowering *MAF2* var1 plants (arrow in Fig. 3c; Supplemental Fig. 2). Cloning and sequencing of bands in the 610–728 bp region (Supplemental Fig. 2) showed that a band at 706 bp, corresponding to *MAF3* var1 is absent in the early flowering, *MAF2* var1 transgenic lines. Therefore, pronounced early flowering in 35S:*MAF2* var1 in T_4_ plants is associated with high transgene expression and suppressed expression of the endogenous *MAF2* gene and some of its paralogues. In summary, the analysis of T_4_ lines linked the early flowering phenotype of *MAF2* var1 transgenics with non-target effects on endogenous *MAF* genes. These non-target effects, as well as the failure of remainder of T_4_ single insert lines to maintain strong over-expression of the transgene across generations prevented examination of the effect of splice variant overexpression on plant phenotypes in Col background.

### *MAF2* var1 partially phenocopies the effect of the *MAF2* genomic fragment in the accession Ll-2

**Fig. 4:**
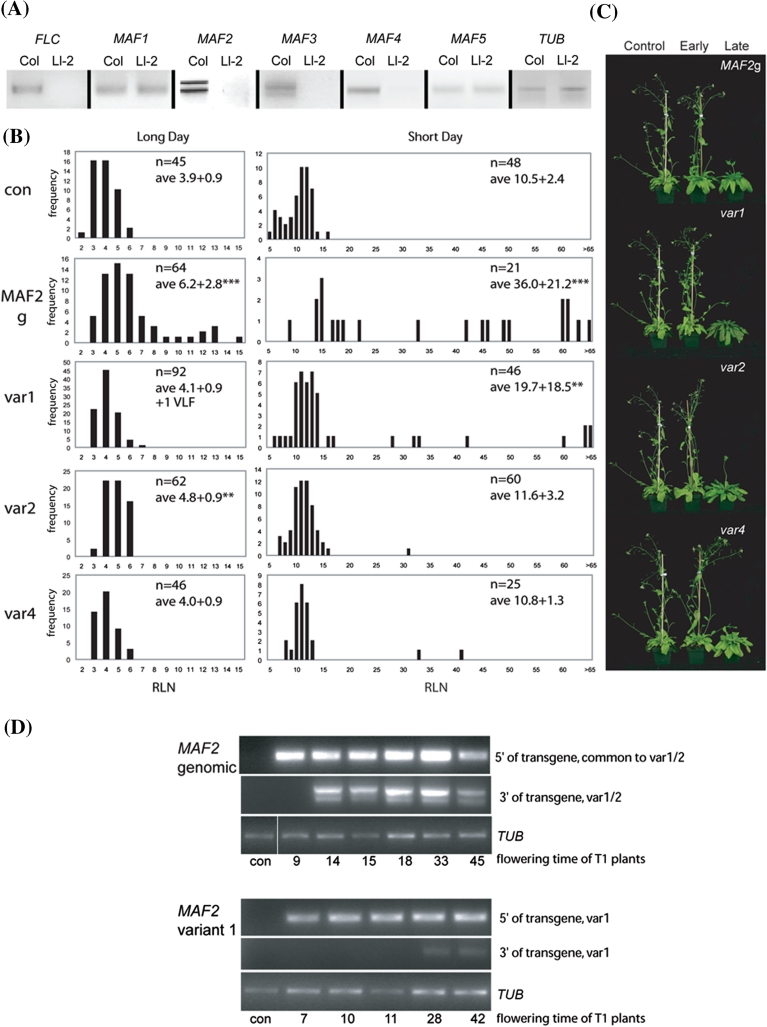
Overexpression of *MAF2* genomic fragment and splice variants in Ll-2 accession. **a** Expression of the *FLC/MAF* gene family in Col and the Ll-2 accessions. **b** Flowering time distribution in T_1_ plants overexpressing *MAF2* genomic fragment (*MAF2g*), *MAF2* var1 (var 1), *MAF2* var2 (var 2) and *MAF2* var4 (var 4). The number of transgenic plants analyzed, the average rosette leaf number (RLN) ± standard deviation and results of an ANOVA test indicating significant differences from the control at * <0.05, ** <0.01, *** <0.001 are shown in the *right hand corner* of each graph. VLF, a late-flowering plant that did not flower during the course of the experiment. **c** Representative transgenic plants from populations shown in b. **d** Analysis of transgene expression in a sample of early and late flowering 35S:*MAF2*g and 35S:*MAF2* var1 T_1_ plants. The 5′ end of cDNA was amplified with primers 5 and 20 and the 3′ end of cDNA was amplified with primers 5 and 6 in 35S:*MAF2*g plants and primers 15 and 16 in 35S:*MAF2* var1 plants (Supplemental Table 1)

Given the confounding effects of strong *MAF2* var1 overexpression on *MAF2*, *MAF3* and *FLC* gene expression leading to the recovery of mostly early flowering transgenic plants, we sought to determine the effect of overexpression of the *MAF2* var1 in plants that do not express *MAF2* and its paralogues. The Ll-2 accession is known to be null for *FLC* (Lempe et al. 2005). In addition, *MAF2* expression was not detectable using primers in the 5′ and 3′-UTR (Fig. 4a) and within the coding region (Rosloski et al. 2010), showing that Ll-2 is also null for *MAF2* expression. While checking the expression of the remaining paralogues, we discovered that Ll-2 also fails to properly express *MAF3* and *MAF4* despite presence of the RT-PCR primer sites at these loci (Fig. 4a). Although genomic DNA sequences of *MAF2*, *MAF3* and *MAF4* in Ll-2 does not give indication as to why expression of these genes is not detectable, the failure of primer sets to detect expression at these loci lend credence to the proposition that Ll-2 is null for wild-type expression of *MAF2* and most of the other clade members. Thus, the Ll-2 accession provides a valuable genetic background in which one could assess the phenotypic effect of overexpression of *MAF2* splice variants without confounding, non-target effects on the endogenous *MAF2* gene and its paralogues.

Ll-2 is an extremely early flowering accession that flowers after producing ~4 rosette leaves under a long-day (LD) photoperiod (Fig. 4b). It was transformed with the same constructs as previously used for Col transformation, and the effect of the transgenes on timing to flowering was determined by counting rosettes leaves (RLN) produced prior to bolting. RLN was determined in T_1_ plants under LD and SD photoperiods at 22 °C (Fig. 4b). Similar to the Col background, overexpression of the *MAF2*g construct in Ll-2 resulted in delayed flowering in both LD and SD day photoperiods. Overexpression of *MAF2* var1 did not affect flowering time of Ll-2 plants grown under LD conditions compared to controls, with the exception of a single, extremely late flowering plant (57 leaves). However, under SD conditions, 35S:*MAF2* var1 plants displayed a similar range of flowering times as seen for the *MAF2*g construct, (Fig. 4b, c) *MAF2* var2 and *MAF2* var4 plants did not flower differently from the controls in SD conditions, though one and two late-flowering plants were observed in these populations, respectively. As *MAF2* var2 and var4 populations were largely undifferentiated from the control we conducted further analysis only on *MAF2g* and *MAF2* var1 populations.

To understand the effect of *MAF2* transgenes on flowering time in Ll-2, we determined the transgene expression in some early and late flowering *MAF2*g and *MAF2* var1 T_1_ plants (Fig. 4d). Transgene expression was determined by two sets of RT-PCR primers: primers 5 and 20, placed at the 5′-end of the transgene, showed that both *MAF2*g and var1 transgenes are overexpressed in transgenic plants; primers 5 and 6 (*MAF2*g) or 15 and 16 (*MAF2* var1), at the 3′-end of the transgene, showed that only a subset of transgenic plants have normal transgene expression by expressing both parts of the transgene. While the mechanism is not known, the frequency of proper expression of *MAF2*g and *MAF2* var1 transgenes associates with the phenotypic distribution of T_1_ lines. Importantly, plants that flower late in both populations of transgenic plants express transgenes properly, suggesting that the late-flowering phenotype under SD conditions is induced by overexpression of *MAF2*g and *MAF2* var1 transgenes.

### Ll-2 plants overexpressing *MAF2* genomic and var1 transgene are weakly responsive to cold treatment

**Fig. 5:**
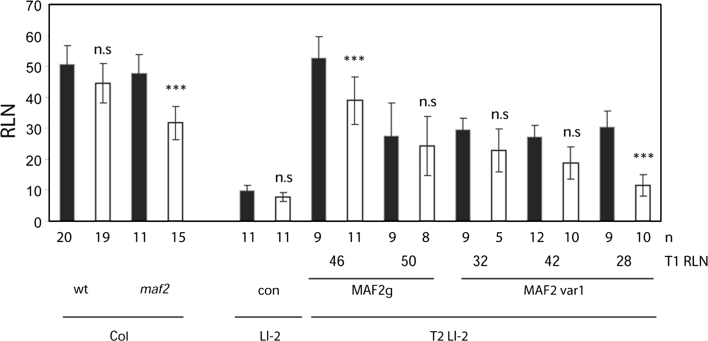
Cold responsiveness of T_2_ Ll-2 plants overexpressing *MAF2* genomic and *MAF2* var1 transgenes. Plants from the Col and Ll-2 backgrounds were selected on 50 mg/L kanamycin for the transgene and either stratified for 3 days (*black bars*) or seedlings were treated with a 16-day cold treatment (*white bars*), and were grown under a short day photoperiod. The flowering time of T_1_ progenitors is shown as the rosette leaf number (RLN) on the x-axis (T_1_ RLN) and the flowering time of T_2_ lines is shown as RLN on the y-axis. Significant differences between the RLN for the 3 and 16 day treatment are shown *above* the 16 day treatment for each pair, * <0.05, ** <0.01, *** <0.001 or n.s. non-significantly different. Sample sizes (n) are shown *under each bar*. Only morphologically normal T_2_ plants were included in the analysis of the flowering time

*MAF2* has been proposed to function as a repressor of flowering during short periods of cold (Ratcliffe et al. 2003). Consistent with this role, the *maf2* T-DNA line is more responsive to cold-induced acceleration of flowering than Col (Fig. 5). Since properly expressed *MAF2*g and var1 transgenes in the Ll-2 background were able to delay flowering under ambient temperature, we tested the response of representative T_2_ transgenic lines to a short period of cold. We have analysed two and three T_2_ transgenic lines expressing *MAF2g* and *MAF2* var1 transgenes, respectively, whose T_1_ progenitor plants flowered in the mid-range of the corresponding T_1_ populations. Under a SD photoperiod, Ll-2 plants flower after producing 9.8 ± 1.8 leaves and are insensitive to vernalization. Like T_1_ progenitor plants, all T_2_ transgenic lines significantly delayed flowering without a cold treatment compared to Ll-2, confirming that both *MAF2*g and *MAF2* var1 transgenes function as repressors of flowering. The ability to delay flowering after a short period of vernalization was variable among the T_2_ lines of both constructs and was weak. Therefore, both *MAF2*g and *MAF2* var1 transgenes are capable of repressing flowering and neither of them have capability to strongly prevent the acceleration of flowering after a moderate period of cold.

## Discussion

Ratcliffe et al. (2003) postulated that *MAF2* functions to prevent flowering after a moderate cold treatment of 16–21 days. This conclusion was based on phenotypic characterization of a T-DNA insertion line that has no detectable *MAF2* transcription and plants overexpressing the full-length cDNA form, *MAF2* var1. Given that the *MAF2* locus generates multiple transcripts, it is unclear if a transcriptionally inactive locus provides insight into the function of an alternatively spliced gene. 35S:*MAF2* var1 T_1_ overexpression plants described in that study were mostly early flowering, although several late flowering plants were also observed (Ratcliffe et al. 2003). The authors concluded that *MAF2* acts as a repressor of flowering based on the heritability of late flowering in advanced transgenic lines. Our study expands the understanding of *MAF2* gene function and the role of the individual splice variants. We show that cold favors expression of *MAF2* var1, the transcript predicted to encode the full-length MADS-box protein, but that it significantly affects the expression of *MAF2* var2, a splice form predicted to encode a truncated MAF2 protein. Our data further reveal that *MAF2* var1 has the greatest ability to delay flowering compared to other observed splice variant forms.

### Cold affects the expression of *MAF2* splice variants

We previously reported that higher ambient temperature affects the expression patterns of *MAF2* (Balasubramanian et al. 2006). Like elevated temperatures, cold treatment affects the abundance of the *MAF2* splice variants, indicating that temperatures at both ends of the spectrum can affect the expression of alternatively spliced genes. However, high and low temperatures affect *MAF2* splicing in different ways: at 25 °C the expression of *MAF2* var2 is favored, while at 4 °C its expression is downregulated (Balasubramanian et al. 2006; Fig. 1c). The retention of *MAF2* var1 expression in the cold renders it as a candidate repressor of flowering during a moderate cold treatment. The inability of the *MAF2* var2 form to delay flowering when overexpressed portrays this splice variant as a non-functional product of transcription (Figs. 2, 3). The protein encoded by the *MAF2* var2 is predicted to have a MADS-box DNA binding domain, but to lack the protein-interaction K-box domain. Given this putative structure, the protein could interfere with the function of the *MAF2* var1 gene product. However, the high potency of the overexpressed *MAF2* genomic clone in flowering repression in Col, *flc*-*3* and Ll-2 backgrounds, giving rise to both *MAF2* var1 and var2 transcripts, suggests that *MAF2* var2 does not impede the functionality of the *MAF2* locus.

Of interest to this study is the series of natural *MAF2* alleles resulting from structural rearrangements that are frequent across the eastern European range of *Arabidopsis thaliana* (Rosloski et al. 2010). These *MAF2* alleles are characterized by large insertions of *MAF3* gene into the *MAF2* coding region. Despite the diversity of rearranged *MAF2* alleles, these alleles are predicted to be incapable of producing *MAF2* var1 proteins but instead produce truncated proteins identical to those predicted from *MAF2* var2, the major wild-type *MAF2* alternative variant, via a premature stop codon. Knock-down of several *MAF3*-insertional alleles had no effect on flowering time, thereby corroborating our assertion that MAF2 var2 protein does not contribute strongly to the repression of flowering (Rosloski et al. 2010).

### Overexpression of *MAF2* var1 in Col affects the expression of the endogenous *MAF2*, *MAF3*, *MAF4* and *FLC* genes

If *MAF2* var1 is the functional repressor of flowering, then this transcript should be able to phenocopy the effect of the overexpression of the *MAF2* genomic fragment. However, similar to Ratcliffe et al. (2003), we found that most T_1_ 35S:*MAF2* var1 in Col plants were very early flowering, failing to support the hypothesis that *MAF2* var1 acts as a floral repressor. However, our analysis of T_4_, single insert transgenic lines in Col showed that the extreme early flowering of the *MAF2* var1 overexpression lines associates with the suppressed expression of the endogenous *MAF2*, *MAF3* and *FLC* genes (Fig. 3b, c). The strongest effect on paralogous MAFs was seen only in plants that robustly expressed *MAF2* var1. Given the high homology of clade members, it is possible that the effect of the *MAF2* var1 transgene on paralogues occurs via a cosuppression-like mechanism, as has been observed during the gene silencing experiments within other gene families (Miki et al. 2005; Travella et al. 2006). It is also possible that artifacts of transgene expression such as truncated transcripts observed in Ll-2, also complicated the analysis of flowering time in Col. Given the high homology of the 5′ *MAF2* gene to paralogues in Col, it is difficult to study the formation of truncated transcripts during overexpression in this accession. However, as extreme early flowering and wide phenotypic distributions have been noted in populations of transgenic plants overexpressing other *MAF* clade genes (Sheldon et al. 1999; Ratcliffe et al. 2001), suppression of *MAF/FLC* paralogue expression in transgenic plants or the formation of truncated transcripts may be significant impediments to the functional analysis of single *MAF* clade genes.

### Overexpression of *MAF2* var1 in Ll-2 represses flowering

To avoid non-target effects of transgene overexpression on endogenous copies of *MAF/FLC* gene family members, we also expressed *MAF2* var1 in the Ll-2 accession that is lacking the expression of the *MAF2*, *MAF3*, *MAF4* and *FLC* genes. Under SD conditions, repression of flowering was observed in the population over-expressing both the *MAF2*g and *MAF2* var1 transgenes. Late flowering transgenic T_1_ plants of both constructs produced late-flowering T_2_ progeny, showing that the effects of these transgenes are stable across generations (Fig. 5).

Early flowering in Ll-2 was associated with the expression of truncated transcripts representing only the 5′-end of each construct. The number of plants expressing truncated transcripts was substantial, representing 17–40 % of T_1_ Ll-2 plants. Plants expressing truncated transcripts were invariably on the early end of the flowering spectrum, showing that transcript truncation generally disrupts the repression of flowering by *MAF2*. *MAF2* var4 is a truncated transcript, being composed only of the MADS-box region of *MAF2*, and, accordingly, 35S:*MAF2* var4 plants rarely displayed late flowering in any population. Because truncated transcripts are predicted to give rise to proteins that retain the DNA binding capability of the MADS-box, they may function to alter the flowering phenotype through non-specific pathways. Of interest, plants expressing truncated transcripts were less frequent in the 35S:*MAF2*g plants, suggesting that the presence of introns may aid either proper transgene insertion or its expression. The presence of truncated transcripts in Ll-2 and the non-target effects in Col recommends analysis of transgenic plant populations for these artifacts when assessing the function of *MAF*-expressing transgenic plants.

There was a distinct effect of day-length on the ability of *MAF2* var1 to delay flowering. In Ll-2, 35S:*MAF2*g consistently repressed flowering under both LD and SD photoperiod, while late flowering was generally not observed in 35S:*MAF2* var1 plants under LD. The *MAF2g* construct contains both exonic and intronic sequences and may be more strongly or stably expressed through a phenomenon termed Intron-Mediated Enhancement (Rose 2002, 2004). The extremely rapid flowering of Ll-2 under LD conditions may necessitate high levels of repressor expression to prevent the initiation of reproduction.

### Temperature-modulated alternative splicing at *MAF2* may be an adaptation of plants to temperature fluctuation

The exposure of plants to cold favors the expression of the *MAF2* var1 that is fully spliced and that is expected to generate the full-length protein. However, the formation of fully spliced transcripts in the cold may not be the norm in *Arabidopsis*. Increased complexity of the splice variant profiles and increased retention of introns is regularly observed during the response of the *Arabidopsis* transcriptome to cold (Ner-Gaon et al. 2004; Robinson and Parkin 2008). The evolution of splice-site temperature optima, differential stability of alternative transcripts in the cold or altered protein–protein interactions at low temperatures might be different ways of altering the splicing efficiency and producing optimal transcripts from genes that function at low temperatures. Similar to *MAF2* var1, expression of the full-length transcript in Black Spruce β-hydroxyacyl ACP dehydratase, a gene product that is likely important for membrane acclimation to low temperatures, is produced only at low temperatures through intron exclusion (Tai et al. 2007). Our data show that *MAF2* var1 has the capability to repress flowering when overexpressed in transgenic plants under ambient temperatures or after a moderate period of cold. The ability of both the *MAF2*g and *MAF2* var1 construct to delay flowering after a moderate period of cold was variable among T_2_ lines and was weak. The weak effect on the vernalization response suggests that *MAF2* may be a locus of minor effect in the vernalization response in comparison to the major effect locus *FLC* (Sánchez-Bermejo et al. 2012; Strange et al. 2011).

It is possible that *MAF2* has functions with more prominent phenotypic outcomes compared to the weak cold response. Expression of the non-functional *MAF2* splice variant, *MAF2* var2, is increased at high temperatures (Balasubramanian et al. 2006). Low temperature decreases expression of the non-functional *MAF2* var2 but does not significantly increase expression of *MAF2* var1, the repressor of flowering (Fig. 1c). Although the phenotypic and gene expression response of *maf2* plants to high temperatures has not been tested quantitatively, alleviation of flowering repression at high temperatures via increased production of a non-functional splice variant may be a more salient response of *MAF2* expression to temperature fluctuation compared to the cold response.

### Gene expression divergence in the *FLC*/*MAF* clade

*FLC* and *MAF2* are paralogous repressors of flowering. The parallel effects of overexpression of *MAF2* splice variants in Col and *flc*-*3* (Fig. 2) indicate that these paralogues affect flowering time independently. In addition, *FLC* and *MAF2* have distinct gene expression patterns in response to cold. *FLC* expression is slowly reduced over a lengthy cold period via epigenetic modulation (He et al. 2004; March-Díaz et al. 2007; Oh et al. 2008), until a low level of *FLC* expression permits the initiation of flowering (Bastow et al. 2004; Sung and Amasino, 2004). Conversely, *MAF2* expression is modulated in two separate ways. Ratcliffe et al. (2003) found that total *MAF2* expression, sampled when plants were returned to 21 °C, was not affected by 3–21 days of cold treatment, but was reduced after 76–85 days of cold. Of possible relevance, Sheldon et al. (2009) demonstrated that repression of *MAF2* expression by a long period of cold was affected by the *VIN3*, *VRN1* and *VRN2* genes that regulate *FLC* epigenetically. Here, we show the second dimension of *MAF2* gene regulation: the temperature-responsive, short to moderate-term alteration of the splice variant profile at 4 °C. This dimension of *MAF2* regulation is distinct from that of *FLC*. Considering the divergence in *MAF2* and *FLC* expression, it will be interesting to discover how the molecular mechanisms of *MAF2* regulation have diverged from those known to control *FLC*, and to determine crucial regulatory elements that allow for the temperature-sensitive alteration of *MAF2* gene expression.

## Electronic supplementary material

Below is the link to the electronic supplementary material.

Supplementary material 1 (DOC 70 kb)

Supplementary material 2 (DOC 30 kb)

Supplemental Fig. 1 Proteins predicted to be expressed in transgenic plants expressing individual splice variant forms (TIFF 3329 kb)

Supplemental Fig. 2 Over expression of MAF2 var1 alters the expression of MAF3. a The gel image of the MAF3 RT-PCR products amplified using primers 7 and 8 (Supplemental Table 1). b Sequencing revealed the identity of bands in the gel region between 610–728 bp and demonstrated that a band at 706 bp, corresponding to MAF3 var1 is absent in the early flowering, MAF2 var1 transgenic lines. c Reported MAF3 splice variants by Ratcliffe et al. (2003) and this study (TIFF 3836 kb)
