# Supplementary material for: Functional analysis of splice variant expression of MADS AFFECTING FLOWERING 2 of Arabidopsis thaliana
Source: Plant Mol Biol. 2012 Oct 31;81(1):57–69. doi: 10.1007/s11103-012-9982-2 (PMC3527738; doi:10.1007/s11103-012-9982-2)
Supplement: Supplementary file 1 — Supplementary material 1 (DOC 70 kb) [file 11103_2012_9982_MOESM1_ESM.doc]

Table S1: Primers and PCR reaction conditions

| **Primer number** | **Primer sequence 5’ to 3’** | **Reference** | **Purpose** | **RT-PCR specifications^a^** |
| --- | --- | --- | --- | --- |
| 1 | GTGAGCTAGGAAGGCAGAACTGA | Scortecci et al., 2001 | Amplification of *MAF1/FLM* cDNA, sqRT-PCR Fig. 3 | 378 bp in Col, 64^o^C, 2 μM, x31 cycles, 1.0% |
| 2 | CCGAAGGAGGTACAACACTGATCC |
| 3 | ACATTGTGGGTCTCCGGTGATTAGGATC | Ratcliffe et al., 2003; Rosloski et al., 2010 | Amplification of *MAF2* cDNA from 5' to 3' UTR in Col, sqRT-PCR, *MAF2* Fig. 1b; *MAF2*-E Fig.3, *MAF2*, Fig. 4a | 702 and 788 bp, 60^o^C, 1.5μM, x27cycles ,1.2-1.4% |
| 4 | AATCAGGCTGTAAGTTTAAGGTGAAAGC |
| 5 | GGCACCAGCCTTATCGGAGG | this paper | Amplification of *MAF2* cDNA in *MAF2*overexpression lines and Col MAF2-T, Fig. 3; 35S:*MAF2*g in Ll-2 sqRT-PCR Fig. 4d. | 505 and 591 bp in Col, 58^o^C, 3μM, x27 cycles, 1.0% |
| 6 | CTCAGCAAGTTCTCCGTTTTTTGAAGG |
| 7 | GAAGAAAAAAAGCAAACACATTTTGGGTCC | Ratcliffe et al., 2003 | Amplification of *MAF3* cDNA from 5' to 3' UTR, sqRT-PCR, Fig. 3 and Fig. 4a | 610 to 786 bp in Col, 56^o^C, 2μM, x31 cycles, 1.6-2.0% |
| 8 | AAGAACTCTGATATTTGTCTACTAAGGTAC |
| 9 | ATTAGGTCAGAAGAATTAGTCGGAGAAAAC | Ratcliffe et al., 2003 | Amplification of *MAF4* cDNA from 5' to 3' UTR, sqRT-PCR, Fig. 3 and Fig. 4a | 765 bp in Col, 60^o^C, 2μM, x31 cycles, 1.0% |
| 10 | CTTGGATGACTTTTCCGTAGCAGGGGGAAG |
| 11 | CACACAAGGAgttactagaaatagttcaaagaaaga | this paper | Amplification of *MAF5* cDNA , sqRT-PCR, Fig. 3 and Fig. 4a | 264 bp in Col, 60^o^C, 2μM, x36 cycles, 1.0% |
| 12 | TTACTGCTCTTTCATCCCCTGTTTCC |
| 13 | GTAGCCGACAAGTCACCTTCT | this paper | Amplification of *FLC* cDNA, sqRT-PCR Fig.3 and Fig. 4a | 569 bp in Col, 58^o^C, 3μM, x27 cycles, 1.0% |
| 14 | TCAACCGCCGATTTAAGGTGGCT |
| 15 | TAGAAATAGTCCAAAGCAAGCTTGAAGAATC | this paper | Amplification of *MAF2* var1 cDNA qRT-PCR Fig. 1; *MAF2* var1-T Fig.3; 3’ end expression of 35S:*MAF2* var1 expression in Ll-2 transgenics Fig. 4d. | qRT-PCR: 527 bp in Col and Ler, 60^o^C, 3μM, 0.3 ul of a 100x SyBR green stock solution in DMSO/25μL reaction, expression monitored over at least 40 cycles  sqRT-PCR: 58^o^C, 3μM, x30 cycles, 1% |
| 16 | CGTCTACGAAGGTACAATAAAGATCTACTAT |
| 17 | TAGCACAAAGACACTTTTATCTCCCTC | Rosloski et al., 2010 | Amplification of *MAF2* var2 cDNA, qRT-PCR Fig. 1c, Fig 3. | qRT-PCR: 296 bp in Col and Ler, 60^o^C, 3μM, 0.3 ul of a 100x SyBR green stock solution in DMSO/25μL reaction, expression monitored over at least 40 cycles  sqRT-PCR: 58^o^C, 3μM, x28 cycles, 1% |
| 18 | CTATAACCAGAAACGTCTTCTTCCC |
| 5 | GGCACCAGCCTTATCGGAGG | this paper | Amplification of expression from the 5’end of *MAF2* Figure 4d; *MAF2*, qRT-PCR Fig. 2 and Supplemental Table 2, column A | 265 bp in Col, 55^o^C, 4μM, x32 cycles, 1% |
| 20 | GTAACGATCAATGATCTTTGACATGTTG |
| 21 | TGTCTCCAAGGGTTCCAGGTT | Rosloski et al., 2010 | Amplification of *TUB2* as loading control, sqRT-PCR  Fig. 1b, Fig. 3, Fig 4d | 920 bp, 60^o^C, 1μM, x22 cycles, 0.8% |
| 22 | TCACCTTCTTCATCCGCAGTT |
| 23 | GAGCCTTACAACGCTACTCTGTCTGTC | this paper | amplification of *TUB2* as loading control, qRT-PCR Fig. 1c | 167 bp in Col and Ler, 60^o^C, 3μM, 0.3 ul of a 100x SyBR green stock solution in DMSO/25μL reaction, expression monitored over at least 40 cycles |
| 24 | ACACCAGACATAGTAGCAGAAATCAAG |
| 25 | AATAGTCCAAAGCAAGCTTG | this paper | Amplification of *MAF2* 3’end, qRT-PCR Supplemental Table 2, column B |  |
| 26 | AATCTTATATCGTCTACGAAGGTAC |

^a^ sqRT-PCR specifications include: expected band size (bp), annealing temperature, MgCl_2_ concentration, cycle number, % agarose for band resolution. qRT-PCR specifications include: expected band size (bp), annealing temperature, MgCl_2_ concentration, minimum cycle number, 0.3 μL SyBR Green dye (Invitrogen) was added to RT-PCR cocktail
