# Supplementary material for: Functional analysis of splice variant expression of MADS AFFECTING FLOWERING 2 of Arabidopsis thaliana
Source: Plant Mol Biol. 2012 Oct 31;81(1):57–69. doi: 10.1007/s11103-012-9982-2 (PMC3527738; doi:10.1007/s11103-012-9982-2)
Supplement: Supplementary file 2 — Supplementary material 2 (DOC 30 kb) [file 11103_2012_9982_MOESM2_ESM.doc]

**Supplemental Table 2.** qRT-PCR of transgene expression from individual T_1_ Col *MAF2* var1 plants. Primers 5 and 20 (column A) or 25 and 26 (column B) were used for qRT-PCR amplification of the 5’ or 3’ end of *MAF2* var1, respectively, from the endogenous *MAF2* gene and the *MAF2* var1 transgene (pimers in Supplemental Table 1). Expression values are relative to Col and result from the average of 3 technical replicates

| **T1 plant** | **RLN** | **Fold change-A^1^** | **Fold change-B^2^** |
| --- | --- | --- | --- |
| OX 67 | 9 | 7.34 | 7.38 |
| OX 68 | 9 | 15.27 | 13.24 |
| OX 69 | 11 | 64.60 | 73.78 |
| OX 70 | 15 | 29.28 | 21.79 |
| OX 71 | 17 | 426.60 | 342.51 |
| WT 22 | 30 | 1.00 | 1.00 |

^1^Expression assayed using primers 25 and 26, and ^2^using primers 27 an 28, Supplemental Table 1
