# Supplementary figures and images for: Functional analysis of splice variant expression of MADS AFFECTING FLOWERING 2 of Arabidopsis thaliana
Source: Plant Mol Biol. 2012 Oct 31;81(1):57–69. doi: 10.1007/s11103-012-9982-2 (PMC3527738; doi:10.1007/s11103-012-9982-2)

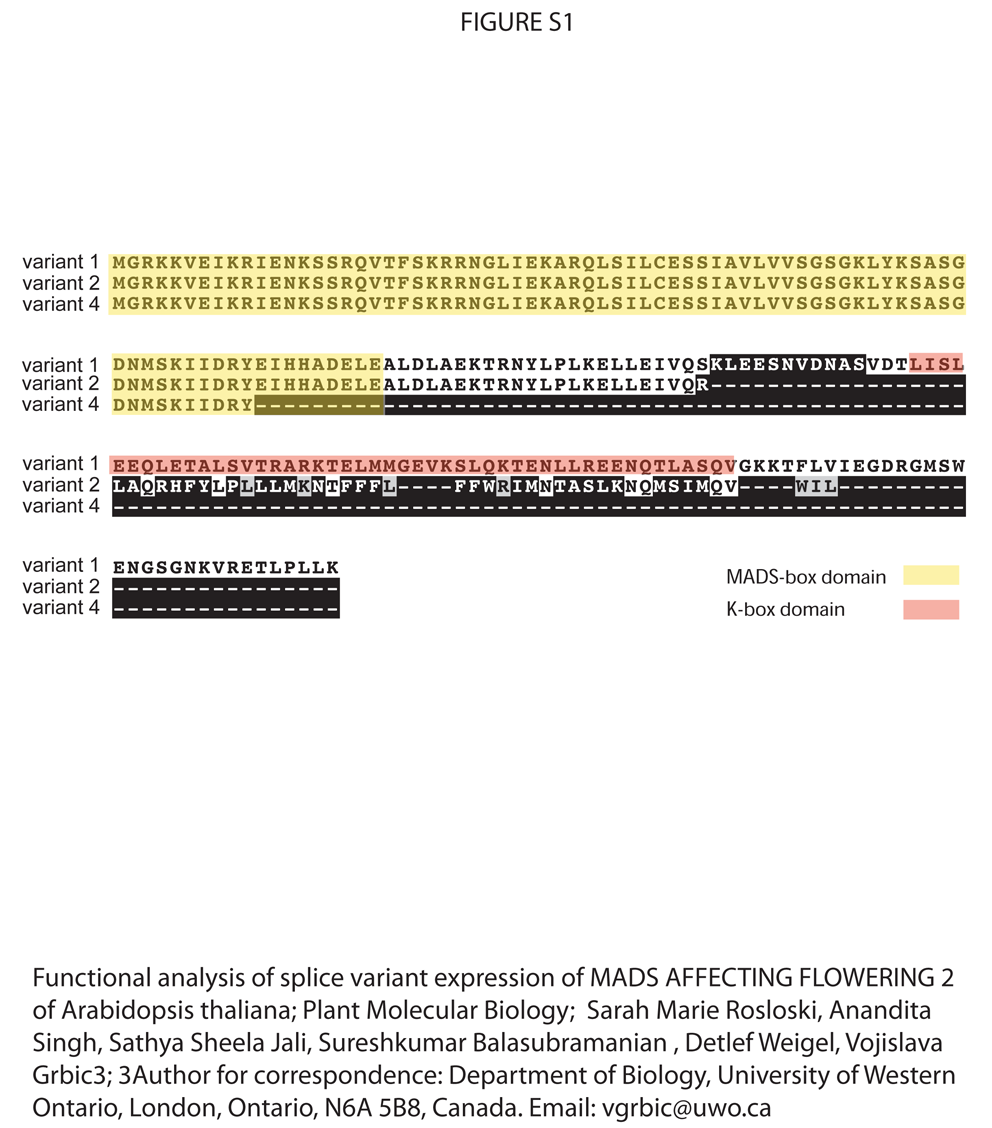

Supplement: Supplementary file 3 — Supplemental Fig. 1 Proteins predicted to be expressed in transgenic plants expressing individual splice variant forms (TIFF 3329 kb) [file 11103_2012_9982_MOESM3_ESM.tif]

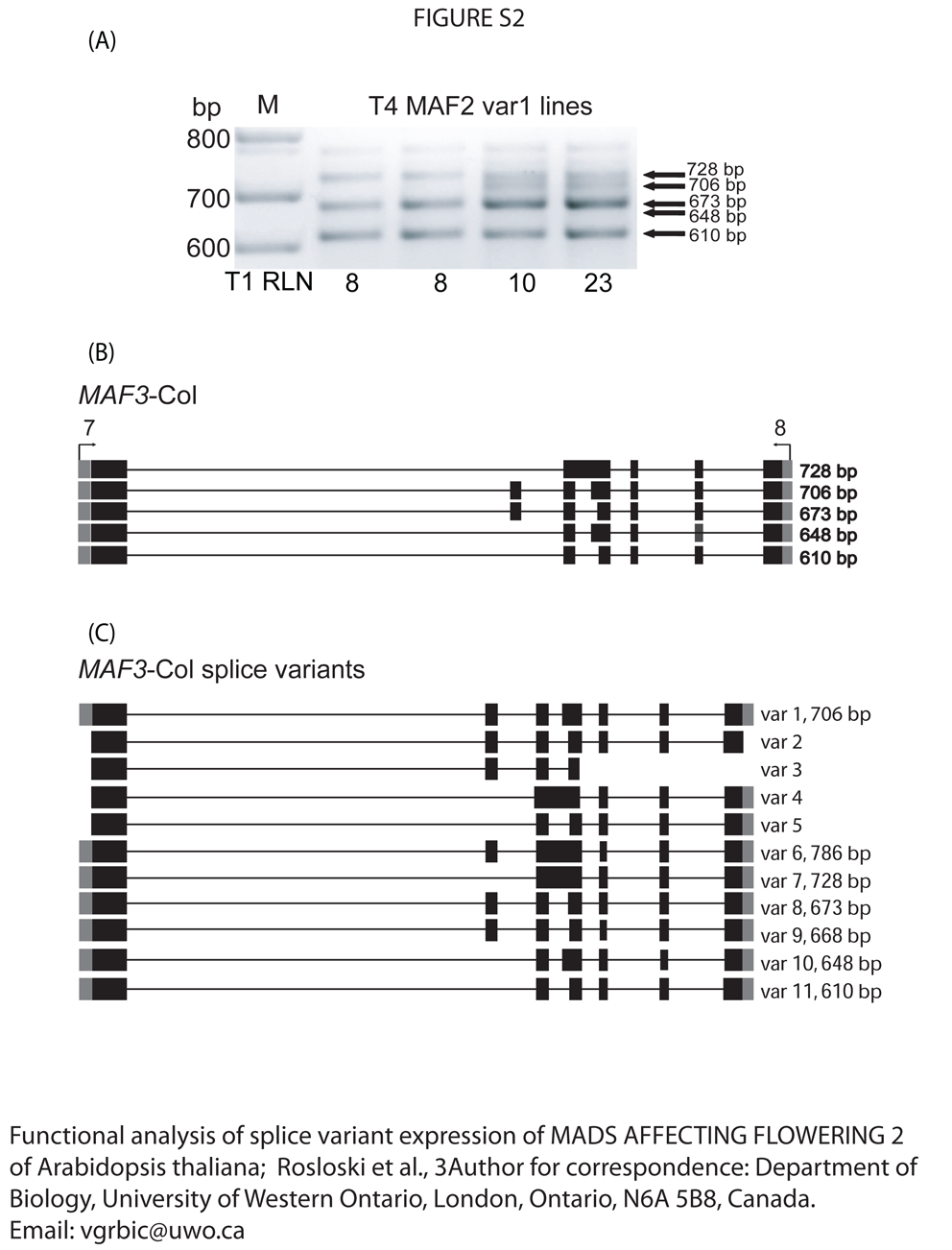

Supplement: Supplementary file 4 — Supplemental Fig. 2 Over expression of MAF2 var1 alters the expression of MAF3. a The gel image of the MAF3 RT-PCR products amplified using primers 7 and 8 (Supplemental Table 1). b Sequencing revealed the identity of bands in the gel region between 610–728 bp and demonstrated that a band at 706 bp, corresponding to MAF3 var1 is absent in the early flowering, MAF2 var1 transgenic lines. c Reported MAF3 splice variants by Ratcliffe et al. (2003) and this study (TIFF 3836 kb) [file 11103_2012_9982_MOESM4_ESM.tif]
